# Editorial: From classical breeding to modern biotechnological advancement in horticultural crops - trait improvement and stress resilience

**DOI:** 10.3389/fpls.2023.1293682

**Published:** 2023-10-11

**Authors:** Pankaj Kumar, Mohammad Irfan, Mohammed Wasim Siddiqui, Radhakrishnan T, Weibiao Liao

**Affiliations:** ^1^ Department of Biotechnology, Dr. Yashwant Singh Parmar University of Horticulture and Forestry, Nauni, Solan, India; ^2^ Plant Biology Section, School of Integrative Plant Science, Cornell University, Ithaca, NY, United States; ^3^ Department of Food Science and Postharvest Technology, Bihar Agricultural University, Bhagalpur, India; ^4^ Indian Council of Agricultural Research-Directorate of Groundnut Research (ICAR-DGR), Junagadh, India; ^5^ College of Horticulture, Gansu Agricultural University, Lanzhou, China

**Keywords:** fruit, vegetable, medicinal plant, horticulture, ornamental plant, antioxidants, stress resilience, biotechnology

Horticultural crops including fruits, vegetables, medicinal, aromatic, and ornamental plants, play a crucial role in enhancing human nutrition and our living environment. Fruits and vegetables are rich sources of essential nutrients, while ornamental plants contribute to aesthetic value. However, losses in quantity and quality during pre and post-harvest stages, as well as the impact of environmental stresses, limit their potential (Irfan et al., 2023). Therefore, developing strategies to improve crop quality and resilience is vital for sustainable production. Traditional breeding methods such as hybridization and mutation breeding have been employed for horticultural crop improvement, but they are time-consuming and labor-intensive, hindering their global adoption in sustainable agriculture. The emergence of molecular genetics has opened doors to modern biotechnological tools for enhancing horticultural crops. In recent decades, genetic engineering and molecular breeding technologies have been extensively used to create horticultural varieties with improved traits (Kumari et al., 2020).

The primary objective of this Research Topic was to offer the most up-to-date research in this field, achieved by gathering contributions from leading experts. This Research Topic comprises 11 papers, consisting of 10 research articles and one review article, collectively exploring various facets of the research theme. To better understand their distinct research trajectories, these articles have been meticulously summarized.

The evolution of horticultural crop improvement has undergone a remarkable transformation, transitioning from classical breeding methods to cutting-edge biotechnological advancements. This progression has paved the way for enhancing traits and stress resilience in these crops. One avenue of advancement lies in the application of biostimulants, which have proven effective in extending the post-harvest longevity of the magnificent bird of paradise cut flower. Thakur et al. investigated the use of graphene oxide (GO) and silver nanoparticles (SNPs) as biostimulants to enhance the post-harvest longevity of bird of paradise (*Strelitzia reginae* L.) cut flowers. The combination of GO and SNPs at a concentration of 1.0 µL L^−1^ significantly extended the vase life of the flowers by six days compared to the control. This improvement was attributed to better water uptake, reduced microbial density, delayed stem blockage, decreased electrolyte leakage, and enhanced antioxidant enzyme activity. These findings suggest that biologically synthesized nanoparticles have potential as a novel post-harvest technology, which holds promise for widespread commercial implementation within the cut flower industry.

Modern biotechnology has also enabled a deeper exploration of the regulatory genes/transcription factors, employing advanced bioinformatics strategies to unravel their biological functions. Gangwar et al. conducted a study on myo-inositol-1-phosphate synthase (MIPS) genes in Rosaceae plants, revealing their importance in various plant processes like signal transduction, stress tolerance, and growth. They identified 51 MIPS genes in 26 Rosaceae species and found them to be conserved in structure and function. The study highlighted the involvement of the *RcMIPS* gene in plant development and its response to abiotic stresses like drought and heat, providing valuable insights for future functional studies in this plant family. Additionally, Ali et al. investigated the impact of boron application on sugar and acid profiles in loquat fruits. This research delves into the intricate physiological, biochemical, and molecular mechanisms that govern the biosynthesis of soluble sugars and malic acid in loquats, with potential applications for fruit quality improvement.

Another noteworthy accomplishment in this realm involves the identification of a naturally anethole-rich strain of *Clausena heptaphylla* through rigorous selection processes, subsequently confirmed *via* multi-location trials (Lal et al.). Twenty-three accessions of anethole were evaluated which ultimately resulted in identifying a superior strain (Jor Lab CH-2) with consistent performance across multiple locations. This strain exhibited an average herbage yield of 1.2 Kg/plant/cutting and an essential oil yield of 1.22%, with trans-anethole as the major constituent (93.25%). This offers a novel and cost-effective alternative for anethole production, expanding industry possibilities. Expanding the horizon, global whole-genome comparisons and analysis have been instrumental in categorizing sub-populations and pinpointing resistance genes within weedy rice, thereby contributing to crop enhancement endeavors. Among the notable findings is the presence of the *RPW8* domain in ORUFILM12g000772, holding potential for plant resistance to pathogens and its applicability in rice breeding programs (Han et al.).

Furthermore, the mechanism underlying nitric oxide-induced adventitious root development under cadmium stress has been thoroughly investigated, shedding light on strategies for stress resilience (Niu et al.). It was reported that nitric oxide (NO), supplied through a donor sodium nitroprusside (SNP), significantly increased the number and length of adventitious roots while also boosting endogenous NO levels. Additionally, NO-enhanced antioxidant defenses reduced oxidative damage, and upregulated the glycolysis and polyamine-related genes. However, the inhibitory effects of NO scavenger and inhibitor suggested a direct link between NO and adventitious root development under Cd stress (Niu et al.). Further studies have explored diverse germplasm lines (186 lines) of *Solanum khasianum* for stability in economically important traits such as solasodine content and fruit yield. These findings have a pivotal role in advancing breeding programs aimed at improving crop quality and yield (Begum et al.).

Li et al. employed inter simple sequence repeats (ISSR) molecular markers and anatomical analysis to screen cold-tolerant mutants of *Plumbago indica* L., a valuable ornamental and medicinal plant. Through tissue culture, chemical treatment, and low-temperature stress induction, a total of nine mutants were identified with altered DNA profiles. These mutants exhibited modified anatomical structures and increased plumbagin content, with four mutants showing enhanced cold resistance. The mutants also demonstrated traits consistent with cold-resistance plants and displayed delayed flowering and senescence. This innovative approach offers promising prospects for the development of cold-resistant varieties in this species. Moreover, Hu et al. have elucidated the role of an R2R3-MYB transcription factor gene (*SmMYB108*) as a transcriptional activator regulating anther development in eggplants. Differentially expressed gene *SmMYB108* in the fertile line F142 across various anther developmental stages has provided profound insights into the molecular mechanisms governing anther development.

The utilization of volatile profiling as a potential biochemical marker has shown promise in validating gamma irradiation-derived putative mutants in polyembryonic genotypes of mango, underscoring its significance in crop improvement strategies (Perveen et al.). It has been reported that volatile profiling *via* headspace-solid phase micro-extraction (HS SPME) method coupled with gas chromatography–mass spectrometry (GC-MS MS) analysis revealed significant differences in major volatile compounds between mutants, control seedlings, and mother plants. Simple sequence repeat (SSR) markers confirmed genetic diversity in the putative mutants, with clustering indicating distinct genetic profiles. This research highlighted the potential of volatile profiling and SSR markers for validating mutations and detecting genetic variation in polyembryonic mango genotypes, particularly when nucellar seedlings closely resemble mother plants (Perveen et al.).

Lastly, the review article by Manzoor et al. emphasizes the limitations of conventional breeding methods for temperate fruit and nut crops, citing factors like long juvenile periods and genetic complexities. They contended that integrating advanced biotechnological tools, such as molecular markers like single-nucleotide polymorphism (SNP), and Diversity Arrays Technology (DArT) markers, can enhance the precision and efficiency of plant selection based on genotypes. These markers enabled the direct targeting of genomic regions governing desired traits, like quality and resistance, saving time and space. Overall, the review highlighted the potential of molecular marker approaches to improve adaptability and stress resistance in temperate fruit and nut species through genome sequencing and cultivar selection.

In summary, the papers published on the aforementioned Research Topic showcase the latest breakthroughs in research. The transition from traditional breeding methods to cutting-edge biotechnological innovations in the realm of horticultural crops has resulted in a treasure trove of knowledge and inventive techniques. These endeavors are all geared towards enhancing characteristics and bolstering stress resistance in these valuable crops. These multifaceted approaches point the way forward for the improvement of horticultural crops, promising heightened sustainability and productivity.

## Author contributions

PK: Writing – original draft, Writing – review & editing. MI: Writing – original draft, Writing – review & editing. MS: Writing – review & editing. RT: Writing – review & editing. WL: Writing – review & editing.
